# Genetic Variants of TSLP and Asthma in an Admixed Urban Population

**DOI:** 10.1371/journal.pone.0025099

**Published:** 2011-09-22

**Authors:** Mengling Liu, Linda Rogers, Qinyi Cheng, Yongzhao Shao, Maria Elena Fernandez-Beros, Joel N. Hirschhorn, Helen N. Lyon, Zofia K. Z. Gajdos, Sailaja Vedantam, Peter Gregersen, Michael F. Seldin, Bertram Bleck, Adaikalavan Ramasamy, Anna-Liisa Hartikainen, Marjo-Riitta Jarvelin, Mikko Kuokkanen, Tarja Laitinen, Johan Eriksson, Terho Lehtimäki, Olli T. Raitakari, Joan Reibman

**Affiliations:** 1 Department of Environmental Medicine, New York University School of Medicine, New York, New York, United States of America; 2 Department of Medicine, New York University School of Medicine, New York, New York, United States of America; 3 Department of Genetics, Harvard Medical School, Boston, Massachusetts, United States of America; 4 Divisions of Genetics and Endocrinology, Children's Hospital, Boston, Massachusetts, United States of America; 5 Broad Institute of Harvard and MIT, Cambridge, Massachusetts, United States of America; 6 Robert S. Boas Center for Genomics and Human Genetics, Feinstein Institute for Medical Research, Manhasset, New York, United States of America; 7 Department of Biochemistry and Molecular Medicine, University of California Davis, Sacramento, California, United States of America; 8 Respiratory Epidemiology and Public Health, Imperial College, London, United Kingdom; 9 Department of Clinical Sciences, Obstetrics and Gynecology, Institute of Clinical Medicine, University of Oulu, Oulu, Finland; 10 Department of Epidemiology and Biostatistics, Imperial College, London, United Kingdom; 11 Department of Chronic Disease Prevention, National Institute for Health and Welfare, Oulu, Finland; 12 Department of Pulmonary Diseases and Clinical Allergology, Turku University Hospital and University of Turku, Turku, Finland; 13 National Institute for Health and Welfare, Finland Department of General Practice and Primary Health Care, University of Helsinki, Finland Helsinki University Central Hospital, Unit of General Practice, Helsinki, Finland Folkhalsan Research Centre, Helsinki, Finland Vasa Central Hospital, Vasa, Finland; 14 Department of Clinical Chemistry, University of Tampere and Tampere University Hospital, Tampere, Finland; 15 Research Centre of Applied and Preventive Medicine, University of Turku and Department of Clinical Physiology, Turku University Hospital, Turku, Finland; University of Medicine and Dentistry of New Jersey, United States of America

## Abstract

**Background:**

Thymic stromal lymphopoietin (TSLP), an IL7-like cytokine produced by bronchial epithelial cells is upregulated in asthma and induces dendritic cell maturation supporting a Th2 response. Environmental pollutants, including tobacco smoke and diesel exhaust particles upregulate TSLP suggesting that TSLP may be an interface between environmental pollution and immune responses in asthma. Since asthma is prevalent in urban communities, variants in the TSLP gene may be important in asthma susceptibility in these populations.

**Objectives:**

To determine whether genetic variants in TSLP are associated with asthma in an urban admixed population.

**Methodology and Main Results:**

Ten tag-SNPs in the TSLP gene were analyzed for association with asthma using 387 clinically diagnosed asthmatic cases and 212 healthy controls from an urban admixed population. One SNP (rs1898671) showed nominally significant association with asthma (odds ratio (OR) = 1.50; 95% confidence interval (95% CI): 1.09–2.05, p = 0.01) after adjusting for age, BMI, income, education and population stratification. Association results were consistent using two different approaches to adjust for population stratification. When stratified by smoking status, the same SNP showed a significantly increased risk associated with asthma in ex-smokers (OR = 2.00, 95% CI: 1.04–3.83, p = 0.04) but not significant in never-smokers (OR = 1.34; 95% CI: 0.93–1.94, p = 0.11). Haplotype-specific score test indicated that an elevated risk for asthma was associated with a specific haplotype of TSLP involving SNP rs1898671 (OR = 1.58, 95% CI: 1.10–2.27, p = 0.01). Association of this SNP with asthma was confirmed in an independent large population-based cohort consortium study (OR = 1.15, 95% CI: 1.07–1.23, p = 0.0003) and the results stratified by smoking status were also validated (ex-smokers: OR = 1.21, 95% CI: 1.08–1.34, p = 0.003; never-smokers: OR = 1.06, 95% CI: 0.94–1.17, p = 0.33).

**Conclusions:**

Genetic variants in TSLP may contribute to asthma susceptibility in admixed urban populations with a gene and environment interaction.

## Introduction

Environmental insults support an immune milieu that promotes allergic asthma [1]. Epithelial cells, the first targets of inhaled environmental insults such as pollution or tobacco smoke, produce cytokines that modify T cell and inflammatory cell responses. Genetic variants of these cytokines may contribute to the susceptibility to asthma. Furthermore, epithelial cell-derived cytokines may be candidate genes that participate in gene-environmental interactions.

Thymic stromal lymphopoietin (TSLP) has been called a “master switch” of allergic inflammation at the epithelial cell and dendritic cell interface [2]. A member of the IL-7 family of cytokines, TSLP induces maturation of myeloid dendritic cells (mDC) that support Th2 polarization and promotes maintenance of the Th2 memory response [2], [3], [4]. TSLP-treated mDC induce an inflammatory Th2 response that is associated with elevated IL-5, IL-4, IL-13, and TNF-α but low IL-10 [2], [5], [6]. Regulatory T cell function is also downregulated by TLSP [7], allowing a Th2 permissive microenvironment [2], [4], [8], [9].

TSLP is expressed by human epithelial cells [2], [10] and is increased in asthmatic airways [7], [11], [12]. We have reported that diesel exhaust particles (DEP) upregulate TSLP expression in human bronchial epithelial cells in response to oxidative stress and that this epithelial-cell derived TSLP induces the functional maturation and Th2 polarization of dendritic cells (DC) [13], [14]. Tobacco smoke extract also upregulates TSLP expression in the murine lung and in smooth muscle [15], [16]. Recently, TNF- α, IL-4, IL-13, rhinovirus, and dsRNA have also been described to upregulate TSLP in human bronchial epithelial cells [17]. Airway epithelial cell expression of TSLP is both necessary and sufficient for the development of airway inflammation in murine models of antigen-induced asthma [18], [19]. These findings reinforce the potential importance of TSLP and its genetic components in environmental-associated asthma.

The gene for TSLP is located on human chromosome 5q22, near the gene cluster encoding Th-2 cytokines [20], [21]. A sex stratified analysis recently showed that a TSLP polymorphism (rs2289276) was associated with cockroach-specific IgE in Costa Rican females [22]. In a large Canadian population, a SNP (rs1837253) 5.7 kb upstream of the TSLP transcription start site was associated with asthma [23] and the association was replicated in a large consortium study [24]. An additional SNP (rs10062929) of the TSLP gene has been identified in association with eosinophilic esophagitis [25].

Urban populations in the United States have high morbidity and mortality from asthma and are highly exposed to ambient air pollutants such as diesel exhaust, environmental tobacco smoke, and indoor allergens such as those from cockroach [26]. These populations are often of diverse racial and ethnic backgrounds, and thus complex populations for genetic studies. Because of the importance of TSLP as a target for environmental-associated asthma, we examined the association of genetic variants of TSLP with asthma in an admixed urban community using genetic ancestral informative markers to control for population substructure. Furthermore, we validated the findings using independent populations.

## Materials and Methods

### Study Population

Asthmatics and healthy controls were identified from the New York University Bellevue Asthma Registry (NYUBAR) in New York City. This registry was approved by the Institutional Review Board of the New York University School of Medicine and all cases and controls signed informed consent. Cases were referred to the registry by the Bellevue Hospital Center Asthma Clinic and local clinics. Controls were referred by asthma cases and by enlisting individuals directly from the community and from other programs within Bellevue Hospital Center. Subjects were excluded if they were less than 18 years old, were current smokers or had a history of >10 pack-year (p-y) tobacco use, had unstable cardiac disease, uncontrolled hypertension, lung disease other than asthma, or neuromuscular disease. Questionnaires and evaluations were completed for all individuals and participants were ascertained with a diagnosis of asthma by a definition modified from the Collaborative Study on Genetics of Asthma [27]. Because most cases were on medication, bronchial hyperresponsiveness with methacholine challenge testing was not performed. The diagnosis was further confirmed using the published algorithm of Enright et al. [28]. To assemble the case-control study, cases and controls were selected to be genetically unrelated with a case to control ratio of approximately 2 to 1, resulting in 387 unrelated asthmatics and 212 healthy controls.

A replication population included 6 population-based cohorts that are part of the Analysis in Population-based Cohorts of Asthma Traits (APCAT) consortium [29]. Asthma diagnosis in these populations was based on physician diagnosed asthma. Individuals with a diagnosis of COPD, chronic bronchitis, or other lung diseases were excluded from the analysis. Cohorts included: the Helsinki Birth Cohort (HBC: 123 cases, 1533 controls), Health 2000 (H2000: 153 cases, 1841 controls), Finrisk (160 cases, 1705 controls) (includes Finrisk 1992, 1997, 2002 and 2007), the North Finnish Birth Cohort 1966 (NFBC66: 364 cases, 3502 controls), Laseri (119 cases, 1844 controls), and the Framingham Heart Study (FH: 797 cases, 6463 controls) for a total of 1716 cases and 16888 controls. All individuals in the studies provided informed consent and all studies were conducted with the approval of the local ethics committees or institutional review boards.

### Allergy testing and Spirometry

Measurements of total serum IgE (total IgE) and allergen-specific IgE for allergens considered significant for the Northeastern United States were performed in a commercial laboratory for the NYUBAR cohort (Pharmacia ImmunoCAP assay; Quest Diagnostics; Teterboro, NJ). An allergen-specific IgE level >0.35 kilo-international units (kIU)/L was considered positive. Pre- and post-bronchodilator spirometry was performed according to American Thoracic Society guidelines [30] and normal values were obtained from Hankinson et al. [31]. Individuals were on a stable dose of medications for one month prior to study but medications were withheld for 6 hours prior to testing.

### Candidate SNP selection and genotyping

Contiguous single nucleotide polymorphisms (SNPs) in the TSLP region on chromosome 5q were identified in the International Haplotype (HapMap) project (International Haplotype Consortium 2003) using data from the European Americans (CEU) and the West African population (YRI). The program Tagger [32] was used to select representative SNPs from the TSLP gene with high linkage disequilibrium (LD) (minor allele frequency >5% and r^2^≥0.8). Genotyping was performed at the Robert S. Boas Center for Genomics and Human genetics on an Illumina BeadStation 500G Golden Gate custom panel using unamplified DNA extracted from blood. Genotyping reproducibility was verified with duplicates. Ten SNPs in TSLP were successfully genotyped with call rate greater than 99% and minor allele frequency (MAF) greater than 1%. The genotypic information and the Hardy-Weinberg analysis results are summarized in Table S1.

Genome-wide genotyping, quality control measures, and imputation in the replication cohorts (APCAT) has been described previously [29]. Briefly, each cohort was genotyped on a platform containing >500,000 SNPs, and, after quality control the genotypes at >2.2 million genotypes were imputed using the HapMap CEU panel as a reference.

Ancestral informative markers (AIMs) with the maximal absolute difference in allele frequency between ancestral populations were used to differentiate continental origins most likely to be represented in the NYUBAR population including diverse Hispanic ancestry [33]. We genotyped 213 AIMs to adjust for population admixture in associate tests [34], [35].

### Statistical analysis

Genotype frequencies of each SNP were tested for the concordance with Hardy-Weinberg equilibrium (HWE) using Pearson's chi-square test in the overall population and then in the control population using R package HardyWeinberg (http://cran.r-project.org/web/packages/HardyWeinberg/index.html). Two approaches, the principal component analysis (PCA) method [36] and the Bayesian STRUCTURE method (version 2.2.3) [37] were implemented using 213 AIMs to adjust for population stratification. Either the first five principal component scores from the PCA approach or the posterior probabilities from the STRUCTURE approach were included in the association analysis as ancestry covariates to adjust for population stratification.

Single SNP association tests with asthma susceptibility assuming an additive allelic effect were performed using the logistic regression, including covariates of age, BMI, income, education and ancestry covariates. Subgroup analyses stratified by smoking status into ex-smokers and never-smokers were also conducted. To test whether multiple genetic variants from the TSLP gene were associated with asthma, haplotypes were reconstructed using the EM algorithm [38] and the haplotype-specific association were tested using the score test approach based on the generalized linear model [39] using R package haplo.stats (http://cran.r-project.org/web/packages/haplo.stats/index.html). All analyses were performed using R 2.9.1.

For the APCAT cohorts, each study separately performed a single marker association analysis assuming an additive allelic model for the imputed data set consisting of ∼2.2 million SNPs. SNPs with low imputation quality (r^2^<0.3 for MACH) were removed from all studies. Each cohort was stratified into current-smokers, ex-smokers and never-smokers, and the association analysis was further performed within each stratum. The analysis was performed using the logistic regression, adjusted for age, gender and the first ten principal components, and accounting for uncertainty at imputed genotypes. The GEE logistic regression test of the GWAF package in R (http://cran.r-project.org/web/packages/GWAF/) was used to correct for familial relatedness in FHS. Test statistics from individual studies were corrected for inflation using genomic control, and then combined using meta-analysis by combining the regression coefficients and standard errors from each study, implemented in METAL (http://www.sph.umich.edu/csg/abecasis/Metal/index.html).

## Results

### Patient characteristics

A total of 387 unrelated asthmatic patients and 212 unrelated healthy controls were included in the analysis of the NYUBAR population. The demographic and clinical characteristics of the study population are shown in Table 1. A majority of the cases and controls were women and were never-smokers. The average age of the cases was slightly but significantly higher than that of the control group (40.1 vs. 36.1, p = 0.0015) and the average BMI of the cases was significantly higher than the control group (29.9 vs. 27.2, p<0.0001). The self-reported race/ethnicity also differed between cases and controls (p = 0.01). Consistent with a diagnosis of asthma, lung function differed significantly between cases and controls, with reduced % of predicted FEV_1_, FVC and FEV_1_/FVC in cases compared to controls. Cases had a significantly higher total IgE and more cases were atopic.

**Table 1:** Characteristics of NYUBAR case-control study.

	Total	Case	Control	p-value
	(N = 599)	(N = 387)	(N = 212)	
**Gender**, N (%)				0.18
Female	409 (68.3)	272 (70.3)	137 (64.6)	
Male	190 (31.7)	115 (29.7)	75 (35.4)	
**Age**, Mean (SD)	38.7 (13.3)	40.1 (14.0)	36.1 (11.4)	0.0015
**BMI**, Mean (SD)	28.9 (7.0)	29.9 (7.4)	27.2 (5.7)	<0.0001
**Income**, N (%)				<0.0001
<$15,000 per year	203 (33.9)	157 (40.6)	46 (21.6)	
$15,000 to $49,999	179 (29.9)	103 (26.6)	76 (35.8)	
> = $50,000	134 (22.4)	66 (17.0)	68 (32.1)	
NA	83 (13.9)	61 (15.8)	22 (10.3)	
**Education**, Mean (SD)	13.4 (3.9)	12.7 (3.9)	14.6 (3.6)	<0.0001
**Self-reported Race/Ethnicity**, N (%)				0.01
Hispanic	326 (54.4)	228 (58.9)	98 (46.2)	
Non-Hispanic Black	118 (19.7)	68 (17.6)	50 (23.6)	
Non-Hispanic White	155 (25.9)	91 (23.5)	64 (30.2)	
**Smoking Status**, N (%)				0.29
Ex-smokers	158 (26.4)	108 (27.9)	50 (23.6)	
Never-smokers	441 (73.6)	279 (72.1)	162 (76.4)	
**Spirometry**				
Pre-FEV_1_, % predicted	83.7 (16.9)	79.2 (17.6)	91.9 (11.8)	<0.0001
Pre-FVC, % predicted	88.5 (15.0)	86.1 (15.8)	92.9 (12.4)	<0.0001
FEV_1_/FVC	77.2 (8.9)	74.8 (9.6)	81.6 (5.1)	<0.0001
**Total IgE** (geo-mean)	81.9	108.7	49.0	<0.0001
**Atopic status**, N (%)				<0.0001
No	184 (30.7)	89 (23.1)	95 (44.8)	
Yes	414 (69.1)	297 (76.9)	117 (55.2)	

### Population stratification

**Figure 1:**
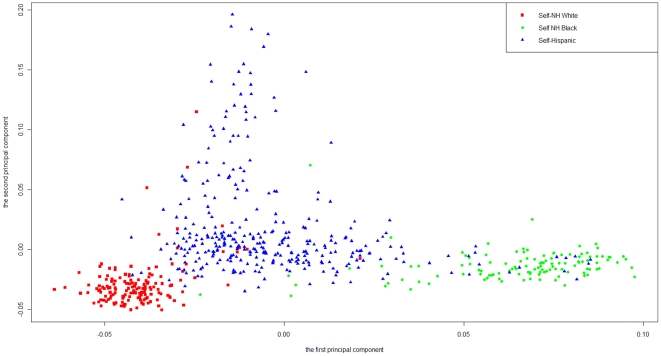
Scatter-plot of the first and second principal components. Scatter-plot of the first and second principal components calculated from the genotypes of 213 AIMs. The first principal component scores showed good separation between the self-reported non-Hispanic white and self-reported non-Hispanic black groups, while the principal component scores of self-reported Hispanic group were in-between.

The first and second principal components from the PCA using 213 AIMs were plotted in Figure 1 with self-reported race/ethnicity information. The first principal component scores showed good separation between the self-reported non-Hispanic white group and the self-reported non-Hispanic black group, while the principal component scores of self-reported Hispanic group were in-between. The first five principal components counted for more than 80% of variability of the ancestry markers. The STRUCTURE method estimates the posterior probability that each subject belongs to each underlying population for each individual. When included as covariates to adjust population stratification in the association tests, the STRUCTURE method resulted in similar results to the PCA method and thus the STRUCTURE results are not reported.

### TSLP and asthma susceptibility

The results of association analysis of asthma and TSLP SNPs are summarized in Table 2. One SNP (rs1898671) was nominally associated with asthma susceptibility in the overall population after adjusting for covariates and population stratification (OR = 1.50, 95% CI: 1.09–2.05, p = 0.01). However, the risk of asthma was increased for this SNP when analyzed in the subgroup of ex-smokers, (OR = 2.00, 95% CI: 1.04–30.83, p = 0.04). In the subgroup of never-smokers, SNP rs1898671 did not show significant association with the risk of asthma (OR = 1.34, 95% CI: 0.93–1.94, p = 0.11).

**Table 2:** Results of association analysis of asthma with TSLP SNPs.

NYUBAR									
dbSNP rs#	Overall			Ex-Smokers			Never Smokers		
	(N = 599)			(N = 158)			(N = 441)		
	OR	95% CI	P	OR	95% CI	P	OR	95% CI	P
rs2289276	0.81	0.61–1.07	0.13	0.89	0.49–1.60	0.69	0.79	0.57–1.08	0.14
rs1898671	1.50	1.09–2.05	0.01	2.00	1.04–3.83	0.04	1.34	0.93–1.94	0.11
rs11466741	0.86	0.66–1.11	0.25	0.78	0.44–1.38	0.39	0.88	0.64–1.19	0.41
rs11466743	1.04	0.47–2.32	0.91	0.80	0.18–3.61	0.77	1.18	0.45–3.08	0.73
rs2289277	0.86	0.67–1.11	0.24	1.05	0.61–1.83	0.85	0.81	0.61–1.09	0.16
rs2289278	0.78	0.52–1.16	0.22	0.73	0.32–1.66	0.46	0.79	0.49–1.27	0.33
rs11241090	0.92	0.51–1.67	0.79	0.94	0.25–3.53	0.92	0.94	0.48–1.83	0.86
rs10035870	1.14	0.63–2.05	0.67	0.52	0.16–1.76	0.30	1.43	0.72–2.85	0.31
rs11466749	1.08	0.73–1.60	0.70	2.28	0.95–5.44	0.06	0.83	0.53–1.32	0.44
rs11466750	0.99	0.70–1.40	0.96	1.81	0.87–3.78	0.11	0.82	0.55–1.23	0.34

APCAT replication									
dbSNP rs#	Overall (N = 18604)			Ex-Smokers* (N = 5872)			Never Smokers* (N = 8060)		
rs1898671	1.15	1.07–1.23	0.0003	1.21	1.08–1.34	0.003	1.06	0.94–1.17	0.33

*: the current smoker group of the APCAT study was not shown.

Because of the suggestion of an association of SNP rs1898671 with asthma, we examined whether the association of this SNP with asthma susceptibility could be replicated in the APCAT cohorts that combined 6 cohorts with 1716 asthma cases and 16888 controls. Analysis of this large cohort revealed consistent results with a positive association between rs1898671 and asthma (OR = 1.15, 95% CI: 1.07–1.23, p = 0.0003). When stratified by smoking history, the replication cohort validated the finding in the NYU cohort that the SNP affected risk in ex-smokers (OR = 1.21, 95% CI: 1.08–1.34, p = 0.003), whereas no significant association was found in never-smokers.

### TSLP haplotype analysis

Reconstructed haplotypes with estimated frequencies of greater than 5% are listed in Table 3 and the estimated haplotype-specific OR with respect to the reference haplotype group (defined as the group with highest frequency) and p-values are also reported. The second most frequent haplotype (GAGGGCAAAG) had an estimated frequency of 24% and showed a significant increased risk with asthma after adjusting age, BMI and population stratification (OR = 1.58, 95% CI: 1.10–2.27, p = 0.01).

**Table 3:** Association results of haplotypes with asthma.

	Haplotype Frequency	Haplotype Specific OR	95% CI of OR	Haplotype Specific P-value
AGAGCCAAAG	0.27	REF		
GAGGGCAAAG	0.24	1.58	1.10–2.27	0.01
GGGGGCAAAG	0.16	1.12	0.75–1.68	0.57
GGGGCCAAGA	0.10	1.19	0.75–1.88	0.47
GGGGGGAAAG	0.09	0.92	0.59–1.46	0.74
GGAGCCAGAG	0.05	1.06	0.63–1.79	0.82
Rare*	0.07	1.24	0.66–2.35	0.50

*:Haplotypes with estimated frequency less than 5% are included in the rare group.

## Discussion

Asthma is prevalent in urban populations of mixed ancestry with high rates of morbidity and mortality in these populations. However, few genetic studies have used diverse urban populations for study because of the complexities of analysis resulting from admixture and mixed ancestry. Moreover, the interaction with environmental exposures may modify asthma risk. TSLP is an epithelial cell-derived cytokine in the IL-7 family that is mechanistically implicated with asthma in numerous human and animal models. Both ambient pollutants and tobacco smoke, common urban environmental exposures, upregulate TSLP [13], [15]. We demonstrated that a SNP variant in TSLP is associated with clinical asthma in an urban population when adjusting for ancestry as well as additional covariates. Examination of an additional large population-based consortium study supported the association between this SNP and clinical asthma. Associations were found to be stronger in those with tobacco exposure. Haplotype analysis of TSLP also revealed an elevated risk of asthma associated with one haplotype. In summary, these data suggest that genetic variants in TSLP may influence asthma risk in complex populations with an environmental interaction.

Our primary population of study was a diverse population and population stratification can influence genetic variation [40], [41], [42], [43]. Indeed, we identified a difference in self-reported race/ethnicity between our asthma cases and controls, suggesting that ancestral differences needed to be accounted for. Moreover a large number of our cases and controls self-reported as Hispanic, a group with complex ancestral history [44]. Thus we accounted for ancestry in our evaluation using SNPs that previously have been associated with similar populations [35] and our results remained consistent even after incorporating ancestry in our evaluation using either of two separate analyses (PCA or STRUCTURE).

The rs1898671 SNP was significant for clinical asthma after adjusting for ancestry and covariates. We did not compensate for multiple testing in these analyses as we considered them discovery testing to be replicated using independent large cohorts. The persistence of significance even after adjustment reinforced the potential importance of this SNP or its associated SNPs. Furthermore, the association of SNP rs1898671 with asthma was replicated in an independent large population-based cohort.

The strength of association of rs1898671 increased when analyzed according to smoking status. The NYUBAR excluded individuals with >10 p-y tobacco use and so examination was limited to those with a less than 10 p-y history of tobacco use. Despite this, the association was strongest in the subgroup with a history of tobacco use. The association with asthma was also stronger in the subgroup with tobacco use in the replication population. This finding suggests a gene-environment interaction effect and is consistent with the studies showing a relationship with TSLP expression and pollutants or tobacco smoke.

The rs1898671 variant is located in an intron and no functional effect of the variant is known. However, we used a tagging algorithm to identify tagSNPs and as such rs1898671 may be in linkage disequilibrium with other SNPs with potential function variation. In the HapMap CEU sample [45], rs1898671 is in strong linkage disequilibrium (r^2^ = 0.91) with rs1043828, which was strongly associated with asthma in the meta-analysis by the GABRIEL consortium (p = 3.1×10−6) [24]. Although the result did not reach genome-wide significance in GABRIEL study, the combination of our results with the published data now strongly suggest that rs1898671 or a variant in LD with rs1898671 influences the susceptibility to asthma. Stratification by smoking status was not available for rs1043828 in the GABRIEL data, so we cannot test whether their association is also stronger in individuals with a history of smoking. We did not perform genotyping for SNP rs1837253 in the NYUBAR cohort but were able to examine the association of this SNP from APCAT cohorts, since this SNP was highlighted by He et al. [23] and the GABRIEL study [24] as possibly associated with severe asthma. The SNP rs1837253 was not significantly associated with asthma risk in the overall population (OR = 1.08, 95% CI: 0.99–1.17, p = 0.1), but did show a nominal association with asthma in Ex-smokers (OR = 1.22, 95% CI: 1.07–1.37, p = 0.01). But this SNP is only in weak LD with rs1898671 (r^2^<0.2), and thus likely represents a separate signal from the associations we observe. Furthermore, the rs1898671 SNP has recently been reported in association with increased risk of eosinophilic esophagitis when comparing to the combined allergic and non-allergic controls [25]. Our exploratory analysis also showed a positive association of this SNP with elevated circulating eosinophils but the result was not statistically significant (data not shown). The possibility exists that this SNP was not detected in other large population studies because of the tobacco interaction.

In summary, we now suggest the association of a TSLP variant and asthma in an admixed population after adjusting for confounders and ancestry. We replicate the finding in an independent population and suggest an interaction with tobacco use. The risk for asthma was associated with a specific haplotype of TSLP involving SNP rs1898671. These data suggest that variants in TSLP may participate in gene and environment interactions associated with asthma susceptibility.

## Supporting Information

Table S1 **Summary of TSLP polymorphisms and Hardy-Weinberg equilibrium test.**
(DOCX)

## References

[1] Saxon A, Diaz-Sanchez D (2005). Air pollution and allergy: you are what you breathe.. Nat Immunol.

[2] Liu YJ, Soumelis V, Watanabe N, Ito T, Wang YH (2007). TSLP: an epithelial cell cytokine that regulates T cell differentiation by conditioning dendritic cell maturation.. Annu Rev Immunol.

[3] Wang YH, Ito T, Wang YH, Homey B, Watanabe N (2006). Maintenance and polarization of human TH2 central memory T cells by thymic stromal lymphopoietin-activated dendritic cells.. Immunity.

[4] Liu YJ (2007). Thymic stromal lymphopoietin and OX40 ligand pathway in the initiation of dendritic cell-mediated allergic inflammation.. J Allergy Clin Immunol.

[5] Ito T, Wang YH, Duramad O, Hori T, Delespesse GJ (2005). TSLP-activated dendritic cells induce an inflammatory T helper type 2 cell response through OX40 ligand.. J Exp Med.

[6] Liu YJ (2006). Thymic stromal lymphopoietin: master switch for allergic inflammation.. J Exp Med.

[7] Nguyen KD, Vanichsarn C, Nadeau KC (2010). TSLP directly impairs pulmonary Treg function: association with aberrant tolerogenic immunity in asthmatic airway.. Allergy Asthma Clin Immunol.

[8] Wang YH, Angkasekwinai P, Lu N, Voo KS, Arima K (2007). IL-25 augments type 2 immune responses by enhancing the expansion and functions of TSLP-DC-activated Th2 memory cells.. J Exp Med.

[9] Valzasina B, Guiducci C, Dislich H, Killeen N, Weinberg AD (2005). Triggering of OX40 (CD134) on CD4(+)CD25+ T cells blocks their inhibitory activity: a novel regulatory role for OX40 and its comparison with GITR.. Blood.

[10] Soumelis V, Reche PA, Kanzler H, Yuan W, Edward G (2002). Human epithelial cells trigger dendritic cell mediated allergic inflammation by producing TSLP.. Nat Immunol.

[11] Ying S, O'Connor B, Ratoff J, Meng Q, Mallett K (2005). Thymic Stromal Lymphopoietin Expression Is Increased in Asthmatic Airways and Correlates with Expression of Th2-Attracting Chemokines and Disease Severity.. J Immunol.

[12] Ying S, O'Connor B, Ratoff J, Meng Q, Fang C (2008). Expression and cellular provenance of thymic stromal lymphopoietin and chemokines in patients with severe asthma and chronic obstructive pulmonary disease.. J Immunol.

[13] Bleck B, Tse DB, Curotto de Lafaille MA, Zhang F, Reibman J (2008). Diesel Exhaust Particle-Exposed Human Bronchial Epithelial Cells Induce Dendritic Cell Maturation and Polarization via Thymic Stromal Lymphopoietin.. J Clin Immunol.

[14] Bleck B, Tse DB, Gordon T, Ahsan MR, Reibman J (2010). Diesel Exhaust Particle-Treated Human Bronchial Epithelial Cells Upregulate Jagged-1 and OX40 Ligand in Myeloid Dendritic Cells via Thymic Stromal Lymphopoietin.. J Immunol.

[15] Nakamura Y, Miyata M, Ohba T, Ando T, Hatsushika K (2008). Cigarette smoke extract induces thymic stromal lymphopoietin expression, leading to T(H)2-type immune responses and airway inflammation.. J Allergy Clin Immunol.

[16] Smelter DF, Sathish V, Thompson MA, Pabelick CM, Vassallo R (2010). Thymic stromal lymphopoietin in cigarette smoke-exposed human airway smooth muscle.. J Immunol.

[17] Kato A, Favoreto S, Avila PC, Schleimer RP (2007). TLR3- and Th2 cytokine-dependent production of thymic stromal lymphopoietin in human airway epithelial cells.. J Immunol.

[18] Zhou B, Comeau MR, De Smedt T, Liggitt HD, Dahl ME (2005). Thymic stromal lymphopoietin as a key initiator of allergic airway inflammation in mice.. Nat Immunol.

[19] Al-Shami A, Spolski R, Kelly J, Keane-Myers A, Leonard WJ (2005). A role for TSLP in the development of inflammation in an asthma model.. J Exp Med.

[20] Reche PA, Soumelis V, Gorman DM, Clifford T, Liu M (2001). Human thymic stromal lymphopoietin preferentially stimulates myeloid cells.. J Immunol.

[21] Quentmeier H, Drexler HG, Fleckenstein D, Zaborski M, Armstrong A (2001). Cloning of human thymic stromal lymphopoietin (TSLP) and signaling mechanisms leading to proliferation.. Leukemia.

[22] Hunninghake GM, Lasky-Su J, Soto-Quiros ME, Avila L, Liang C (2008). Sex-stratified linkage analysis identifies a female-specific locus for IgE to cockroach in Costa Ricans.. Am J Respir Crit Care Med.

[23] He JQ, Hallstrand TS, Knight D, Chan-Yeung M, Sandford A (2009). A thymic stromal lymphopoietin gene variant is associated with asthma and airway hyperresponsiveness.. J Allergy Clin Immunol.

[24] Moffatt MF, Gut IG, Demenais F, Strachan DP, Bouzigon E (2010). A large-scale, consortium-based genomewide association study of asthma.. N Engl J Med.

[25] Sherrill JD, Gao PS, Stucke EM, Blanchard C, Collins MH (2010). Variants of thymic stromal lymphopoietin and its receptor associate with eosinophilic esophagitis.. J Allergy Clin Immunol.

[26] Gold DR, Wright R (2005). Population disparities in asthma.. Annu Rev Public Health.

[27] Meyers DA, Wjst M, Ober C (2001). Description of three data sets: Collaborative Study on the Genetics of Asthma (CSGA), the German Affected-Sib-Pair Study, and the Hutterites of South Dakota.. Genet Epidemiol.

[28] Enright PL, McClelland RL, Newman AB, Gottlieb DJ, Lebowitz MD (1999). Underdiagnosis and undertreatment of asthma in the elderly. Cardiovascular Health Study Research Group.. Chest.

[29] Widen E, Ripatti S, Cousminer DL, Surakka I, Lappalainen T (2010). Distinct variants at LIN28B influence growth in height from birth to adulthood.. Am J Hum Genet.

[30] ATS (1995). Standardization of Spirometry, 1994 Update. American Thoracic Society.. Am J Respir Crit Care Med.

[31] Hankinson JL, Odencrantz JR, Fedan KB (1999). Spirometric reference values from a sample of the general U.S. population.. Am J Respir Crit Care Med.

[32] de Bakker PI, Yelensky R, Pe'er I, Gabriel SB, Daly MJ (2005). Efficiency and power in genetic association studies.. Nat Genet.

[33] Kosoy R, Nassir R, Tian C, White PA, Butler LM (2009). Ancestry informative marker sets for determining continental origin and admixture proportions in common populations in America.. Hum Mutat.

[34] Collins-Schramm HE, Chima B, Morii T, Wah K, Figueroa Y (2004). Mexican American ancestry-informative markers: examination of population structure and marker characteristics in European Americans, Mexican Americans, Amerindians and Asians.. Hum Genet.

[35] Yang N, Li H, Criswell LA, Gregersen PK, Alarcon-Riquelme ME (2005). Examination of ancestry and ethnic affiliation using highly informative diallelic DNA markers: application to diverse and admixed populations and implications for clinical epidemiology and forensic medicine.. Hum Genet.

[36] Price AL, Patterson NJ, Plenge RM, Weinblatt ME, Shadick NA (2006). Principal components analysis corrects for stratification in genome-wide association studies.. Nat Genet.

[37] Pritchard JK, Stephens M, Rosenberg NA, Donnelly P (2000). Association mapping in structured populations.. Am J Hum Genet.

[38] Excoffier L, Slatkin M (1995). Maximum-likelihood estimation of molecular haplotype frequencies in a diploid population.. Mol Biol Evol.

[39] Schaid DJ, Rowland CM, Tines DE, Jacobson RM, Poland GA (2002). Score tests for association between traits and haplotypes when linkage phase is ambiguous.. Am J Hum Genet.

[40] Rotimi CN, Jorde LB (2010). Ancestry and disease in the age of genomic medicine.. N Engl J Med.

[41] Tian C, Gregersen PK, Seldin MF (2008). Accounting for ancestry: population substructure and genome-wide association studies.. Hum Mol Genet.

[42] Caulfield T, Fullerton SM, Ali-Khan SE, Arbour L, Burchard EG (2009). Race and ancestry in biomedical research: exploring the challenges.. Genome Med.

[43] Shriver MD, Smith MW, Jin L, Marcini A, Akey JM (1997). Ethnic-affiliation estimation by use of population-specific DNA markers.. Am J Hum Genet.

[44] Hunninghake GM, Weiss ST, Celedon JC (2006). Asthma in Hispanics.. Am J Respir Crit Care Med.

[45] The International HapMap Consortium (2003). The International HapMap Project.. Nature.

