# Supplementary material for: Genetic Variants of TSLP and Asthma in an Admixed Urban Population
Source: PLoS One. 2011 Sep 22;6(9):e25099. doi: 10.1371/journal.pone.0025099 (PMC3178593; doi:10.1371/journal.pone.0025099)
Supplement: Table S1 — Summary of TSLP polymorphisms and Hardy-Weinberg equilibrium test. (DOCX) [file pone.0025099.s001.docx]

Table S1: Summary of TSLP polymorphisms and Hardy-Weinberg equilibrium test.

| dbSNP rs# | SNP Location | Chromosomal location | Nucleotide Change | Genotype | Genotype Frequency | MAF | HWE P-value |
| --- | --- | --- | --- | --- | --- | --- | --- |
| rs2289276 | Exon 1 | 110435406 | G/A | GG\|AG\|AA | 313\|233\|53 | 0.28 | 0.35 |
| rs1898671 | Intron 1 | 110435901 | G/A | GG\|AG\|AA | 347\|213\|39 | 0.24 | 0.47 |
| rs11466741 | Intron 2 boundary | 110436604 | G/A | GG\|AG\|AA | 268\|260\|71 | 0.34 | 0.56 |
| rs11466743 | Intron 2 | 110436766 | G/A | GG\|AG\|AA | 569\|29\|1 | 0.03 | 0.84 |
| rs2289277 | Intron 2 | 110436966 | G/C | GG\|CG\|CC | 184\|295\|120 | 0.45 | 0.97 |
| rs2289278 | Intron 2 | 110437047 | C/G | CC\|CG\|GG | 492\|96\|9 | 0.10 | 0.14 |
| rs11241090 | Intron 3 | 110437565 | A/G | AA\|AG\|GG | 544\|50\|3 | 0.05 | 0.27 |
| rs10035870 | Intron 3 | 110438763 | A/G | AA\|AG\|GG | 539\|56\|3 | 0.05 | 0.44 |
| rs11466749 | 3’UTR | 110440484 | A/G | AA\|AG\|GG | 470\|119\|9 | 0.11 | 0.77 |
| rs11466750 | 3’ UTR | 110440793 | G/A | GG\|AG\|AA | 439\|142\|18 | 0.15 | 0.16 |
